# Regulation of the Tumor Suppressor PTEN through Exosomes: A Diagnostic Potential for Prostate Cancer

**DOI:** 10.1371/journal.pone.0070047

**Published:** 2013-07-25

**Authors:** Kathleen Gabriel, Alistair Ingram, Richard Austin, Anil Kapoor, Damu Tang, Fadwa Majeed, Talha Qureshi, Khalid Al-Nedawi

**Affiliations:** 1 Division of Nephrology, Department of Medicine, McMaster University, Hamilton, Ontario, Canada; 2 Hamilton Centre for Kidney Research (HCKR), St. Joseph’s Hospital, Hamilton, Ontario, Canada; 3 Division of Urology, Department of Surgery, McMaster University, Hamilton, Ontario, Canada; University of Nebraska Medical Center, United States of America

## Abstract

PTEN is a potent tumor-suppressor protein. Aggressive and metastatic prostate cancer (PC) is associated with a reduction or loss of PTEN expression. PTEN reduction often occurs without gene mutations, and its downregulation is not fully understood. Herein, we show that PTEN is incorporated in the cargo of exosomes derived from cancer cells. PTEN is not detected in exosomes derived from normal, noncancerous cells. We found that PTEN can be transferred to other cells through exosomes. In cells that have a reduction or complete loss of PTEN expression, the transferred PTEN is competent to confer tumor-suppression activity to acceptor cells. In PC patients, we show that PTEN is incorporated in the cargo of exosomes that circulate in their blood. Interestingly, normal subjects have no PTEN expression in their blood exosomes. Further, we found that the prostate-specific antigen (PSA) is incorporated in PC patients’ and normal subjects’ blood exosomes. These data suggest that exosomal PTEN can compensate for PTEN loss in PTEN deficient cells, and may have diagnostic value for prostate cancer.

## Introduction

Prostate cancer (PC) is the most frequently diagnosed cancer and the second highest cause of cancer-related deaths in men [1], [2], [3]. The loss of one copy of the PTEN gene contributes to prostate tumor initiation, while further reduction in PTEN expression supports the invasion and metastatic behavior of PC [4]. PTEN is a protein/lipid phosphatase. Its protein tyrosine phosphatase domain has the features of a dual-specificity phosphatase that is able to dephosphorylate both tyrosine and serine/threonine residues. The main lipid substrate of PTEN is phosphatidylinositol (3,4,5) triphosphate (PIP-3). The main mechanism of tumor suppression by PTEN is the maintenance of cellular PIP-3 at low levels, thus inhibiting the PI3K-AKT pathway and contributing to cellular apoptosis or cell cycle arrest [5]. The reduction of PTEN protein expression often occurs in the absence of gene mutations [6], [7], [8]. Altogether, approximately 70–80% of primary PC tumors have a reduction in PTEN expression [9]. Different mechanisms contributing to the reduction of PTEN expression in tumors have been identified, including promoter methylation [10], [11], and negative regulator proteins [12]. It has been suggested that other, unknown mechanisms may be acting in many tumors [10], [11]. Our results point to a new mechanism by which cancer cells regulate PTEN expression through exosomes.

Cancer cells release vesicles into their surroundings. Microvesicles are one variety of shed vesicles, generated through the direct budding of the cell membrane. Exosomes are another, relatively smaller type of vesicle which are stored in multivesicular bodies and released when the multivesicular body fuses with the cell membrane [13], [14]. Exosome content reflects its cellular source [15], [16]. Interestingly, these contents might include oncogenic proteins, as we have previously reported [17], [18], or tumor suppressor proteins, as reported herein. Thus, one could anticipate that molecules transferred by exosomes confer an acquired phenotype to acceptor cells, leading to positive or negative effects in relation to tumor progression dependent on the nature of the molecules transferred. The cargo of exosomes might thus alter the balance between oncogenic and tumor suppressor characteristics. The analysis of such cargo could indicate the expression status of tumor suppressor proteins in malignant cells without having to directly sample the malignancy. Exosomes are emerging as an important source for cancer biomarkers and are described as biomarker treasure chests for PC [19]. It has been suggested that PTEN status in PC patients could be a predictor of patients at risk for cancer metastasis or recurrence after radical prostatectomy [20], [21], [22]. For several decades now, prostate-specific antigen (PSA) has been used as the standard biomarker for the detection of PC [23]. However, the use of PSA is limited by its lack of specificity and inability to differentiate between indolent and life-threatening forms of the disease at the time of diagnosis [24]. PSA screening may reduce the mortality rate of PC, but it is also associated with a high rate of overdiagnosis and overtreatment [25], [26]. In their study, Harvey et al. concluded that the PSA test has a high false positive and significant false negative rate [27]. This lack of prognostic value leads to an enormous increase in unnecessary biopsies and in the overtreatment of low-risk PC patients [23], [25]. Given these findings, there is a serious need to find new markers for PC or to enhance the specificity of the PSA test.

## Materials and Methods

### Materials

Monoclonal antibodies for PTEN, AKT, Flotilin-1, p27, and cyclin D1 were purchased from Cell Signaling Technology (Danvers, MA). All the corresponding HRP-conjugated secondary antibodies were purchased from Cell Signaling. Alexa Fluor 488 secondary antibodies were purchased from Molecular Probes (Eugene, OR).

#### Cell lines

DU145, PC-3 (Human prostate cancer cells), U87 (human glioblastoma astrocytoma) and the human normal cells [Human aortic endothelial cells (HAOEC), Human aortic smooth muscles cells (HAOSMC) and Human Prostate Epithelial Cells (HPEC)] were purchased from the ATCC (Manassas, VA). DU145 cells with PTEN knockdown (DU145Kd) and DU145 cells transfected with nonspecific siRNA were originally generated in one of our laboratories (D.T.) at the Hamilton Kidney Research Centre (HKRC), McMaster University. DU145Kd cells were generated using PTEN siRNA expressed by a retroviral-based H1 promoter-driven shRNA vector, and the control DU145 cells were infected with a retrovirus expressing nonspecific siRNA, as previously detailed [12]. CHO and CHO-EGFR cells were obtained previously as a generous gift from Dr Guha’s laboratory at The Hospital for Sick Children, Toronto. All the cell lines used in this study were cultured in microvesicle-depleted FBS (by centrifugation overnight at 100,000 *g*). For standard culture, cells were grown in Dulbecco’s modified essential medium (DMEM) supplemented with 10% fetal bovine serum (FBS).

### The Isolation of Exosomes from the Conditioned Media and Blood Plasma

Exosomes were collected from the media of different cell lines and from human plasma, as previously described [18], [28], [29]. Briefly, conditioned medium was collected from cells at approximately 80% confluence, unless indicated otherwise, and this material was subjected to two consecutive centrifugations at 300 *g* for 5 minutes and then at 12,000 *g* for 20 minutes to eliminate cells and debris. Finally, exosomes were obtained after centrifugation for 2 hours at 100,000 *g* and then washed twice with a large volume of phosphate buffered saline (PBS). This protocol specifically collects exosomes and excludes large vesicles. The exosome proteins recovered were measured using the Bradford assay (Bio-Rad).

### PTEN Expression Profile

The cells (DU145, DU145Kd, and DU145 with control siRNA) and the primary cells (HAOEC, HAOSMC and HPEC), along with their corresponding exosomes, were lysed for 10 minutes on ice in a lysis buffer containing: 10 mM Tris (pH 6.8), 5 mM EDTA, 50 mM NaF, 30 mM sodium pyrophosphate, 2% (wt./vol) SDS, 1 mM phenylmethylsulfonyl fluoride (PMSF), and 1 mM Na_3_VO_4_. The lysates were resolved by SDS-PAGE and subjected to immunoblotting with rabbit monoclonal antibodies for PTEN. Immunodetection was accomplished using the appropriate HRP-conjugated secondary antibody and chemiluminescence plus kit (ECL kit; Amersham Pharmacia, Buckinghamshire, United Kingdom), after which the blots were scanned and protein bands quantitated using the Quantity One software (Bio-Rad, CA).

### The Detection of Signaling Events Related to PTEN

To assess the impact of exosomes that contain PTEN on DU145Kd cells, the latter were plated in 100 mm dishes at a density of 2×10^5^ cells/mL, grown briefly, and starved for 24 hours (either in DMEM supplemented with 0.5% FBS, or in serum free DMEM). The cultures were then stimulated overnight with different concentrations of exosomes derived from DU145 cells. After exosome treatment, cell lysates were prepared and analyzed for their signaling effector content using anti-phospho-AKT antibodies (Cell Signaling), according to the supplier’s recommendations. The detection of the expression of p27 and cyclin D1 was performed using the same experimental settings used for the detection of pAKT.

### Fluorescent Imaging of Exosome Uptake

For *in vitro* analysis of PTEN expression, the cells were grown in chamber slides (Nalge Nunc, NY). The cultures were washed with PBS and fixed in preheated (37°C) 4% (wt./vol) paraformaldehyde (PFA) in phosphate buffered saline for 5 minutes. Next, they were washed three times in PBS, and antiquenching was performed in 50 mM NH_4_Cl for 10 minutes at room temperature. Subsequently, the cells were washed twice in PBS and incubated with BSA [1% (wt./vol) in PBS] for 30 minutes. Incubation with a primary antibody was performed for 1 h, followed by washing in PBS, and then incubation with a secondary antibody for 30 minutes. After staining, the slides were mounted using Dako fluorescent mounting medium and viewed under a confocal microscope to detect the presence of exosomal content (PTEN) in the recipient cells.

### PTEN Activity Assay

Lysates from DU145Kd cells, which were treated with exosomes from DU145 cells, were subjected to immunoprecipitation using PTEN antibodies (Cell Signaling) and Protein G Sepharose. PTEN activity was assessed using the water-soluble substrate DiC8PtdIns (3, 4, 5) P3 (Echelon). The released free phosphates were measured with BIOMOL Green reagent and normalized against a reaction containing only PIP3 substrate [12].

### Detection of PTEN mRNA

DU145Kd cells were treated with exosome preparations derived from DU145, followed by extensive washing and the extraction of RNA using Trizol reagent (Invitrogen, NY). RT-PCR analysis was performed using a single-step method (Qiagen, CA) where PTEN was detected using the primer sets: sense 50-ATGACAGCCATCATCAAAGAG-30 and antisense 50-GTGCCACTGGTCTATAATCCAG-30 [12]. The reactions were conducted in 50 µL with the initial Taq activation at 95°C for 30 minutes, followed by 30 cycles of denaturation at 95°C for 30 seconds, primer annealing at 60°C for 1 minute, and extension at 72°C for 30 seconds. The products were resolved on 1% agarose gel and photographed. GAPDH was used as internal control using the primer sets: sense 50-TGATGACATCAAGAAGGTGGTGAAG-30 and antisense 50-TCCTTGGAGGCCATGTGGGCCAT-30.

### Proliferation Assay

The proliferation assay was performed using an assay kit (CHEMICON) according to the manufacturer’s instructions. Briefly, 0.1×10^4^ DU145Kd cells were plated in a 96-well plate; after 24 hours, the cells were treated with different concentrations of exosomes derived from DU145 cells. Other DU145Kd cells were treated with DU145Kd-derived exosomes to show the effect of exosomes with downregulated PTEN. DU145 cells were used as a control to show the ability of exosomes to compensate for PTEN downregulation in DU145Kd. This procedure was used for U87 and PC-3 cells.

### Prostate Cancer Patients and Normal Subjects

This study is supported by ethical approval from the McMaster University ethical board (REB # 02-2174). The participants were informed about the purpose of the study, and written consent has been obtained from all individuals who participated in the study. Samples from 30 PC patients were used in this study. The patients had advanced (T3/T4) tumour stage. Blood samples were collected prior to prostatectomy, and tumor tissues were collected after surgery and snap frozen in liquid nitrogen. Clinical records such as Gleason score, PSA, tumor size, tumor histopathological grade and metastasis were collected. Samples were collected according to the McMaster University ethical standards, and patient consent was obtained before sampling. Eight healthy volunteer men aged 50–65 (matching the PC patient samples) were used in this study; all were without any history of cancer and with normal PSA levels.

### Statistical Analysis

All experiments were reproduced at least three times with similar results. The quantitative data are presented as the average value of the replicates within the representative experiment ± SEM. Statistical significance was evaluated using a computerized 2-tailed Student’s t-test. The differences were considered significant at P<0.05.

## Results

### PTEN is Expressed in Exosomes from PC Cells, but not Normal Cells

**Figure 1:**
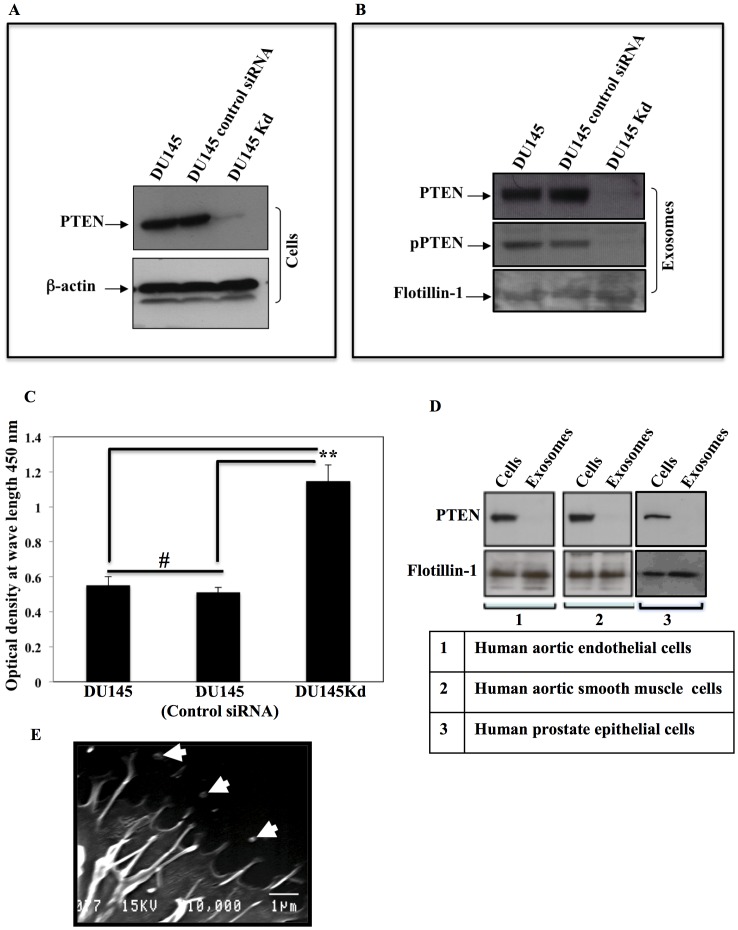
Prostate cancer cells produce exosomes containing PTEN. A. DU145 PC cells were transfected with the indicated siRNA. The cells transfected with PTEN-specific siRNA showed a clear knockdown of PTEN expression, while cells transfected with control mismatch siRNA showed no decrease in PTEN expression. B. Exosomes derived from DU145, DU145Kd, and DU145 with control siRNA show the same pattern of PTEN expression as observed with the cell from which they originated. PTEN incorporated in exosomes is phosphorylated; phosphorylation protects PTEN from degradation, and it can be activated by dephosphorylation upon transfer to other cells. Exosomes are positive for Flotilin-1. C. Differences in proliferation of DU145, DU145 with control siRNA and DU145Kd were assessed, with significantly higher proliferation exhibited by DU145Kd. D. Human primary cells, HAEC, HASMC and HPEC, and their exosomes were profiled for PTEN expression. All three cells have PTEN expression, but PTEN is not expressed in their exosomes. The expression for both cells and their exosomes was normalized with Flotilin-1. E. A scanning electron micrograph showing the shedding of exosomes from DU145 cells.

We determined the status of PTEN in the following PC cells: DU145, DU145 stably transfected with PTEN siRNA (DU145Kd) for PTEN downregulation or knockdown, and DU145 transfected with control siRNA [12]. Exosomes were collected from the conditioned media of each cell type, and equal protein concentrations from the cells and the exosomes were subjected to SDS-PAGE and immunoblotting, then probed with PTEN antibodies. Both DU145Kd cells (Figure 1A) and exosomes (Figure 1B) showed a downregulation of PTEN expression compared to the wild type DU145 cells and DU145 cells transfected with control siRNA. We also assessed the phosphorylation status of PTEN in the exosomes derived from these cells. Phosphorylation of PTEN keeps it in a closed state protected from degradation; later PTEN will be available to be activated in the cytoplasm, bind the cell membrane upon dephosphorylation, and then initiate cell signaling [30], [31]. We found that the PTEN incorporated in exosomes is phosphorylated (Figure 1B, middle panel). Flotilin-1 was used as a loading control for exosomes [32]. The proliferation rates of DU145 parental cells, DU145 with control siRNA and DU145Kd cells (Figure 1C) show that the PTEN knockdown cells exhibited a significantly higher proliferation rate than the other two cell types. Human primary cells (normal, non-cancerous cells), such as Human Aortic Endothelial Cells (HAOEC), Human Aortic Smooth Muscle Cells (HAOSMC), and Human Prostate Epithelial Cells (HPEC) express PTEN, but we found that PTEN is not incorporated into the exosomes of these cells (Figure 1D). This may mean that the incorporation of PTEN in exosomes is an exclusive characteristic of cancer cells, however this finding requires further exploration. Figure 1E is a scanning electron micrograph showing the shedding of exosomes from cancer cells.

### Intercellular Transfer of PTEN by Exosomes

**Figure 2:**
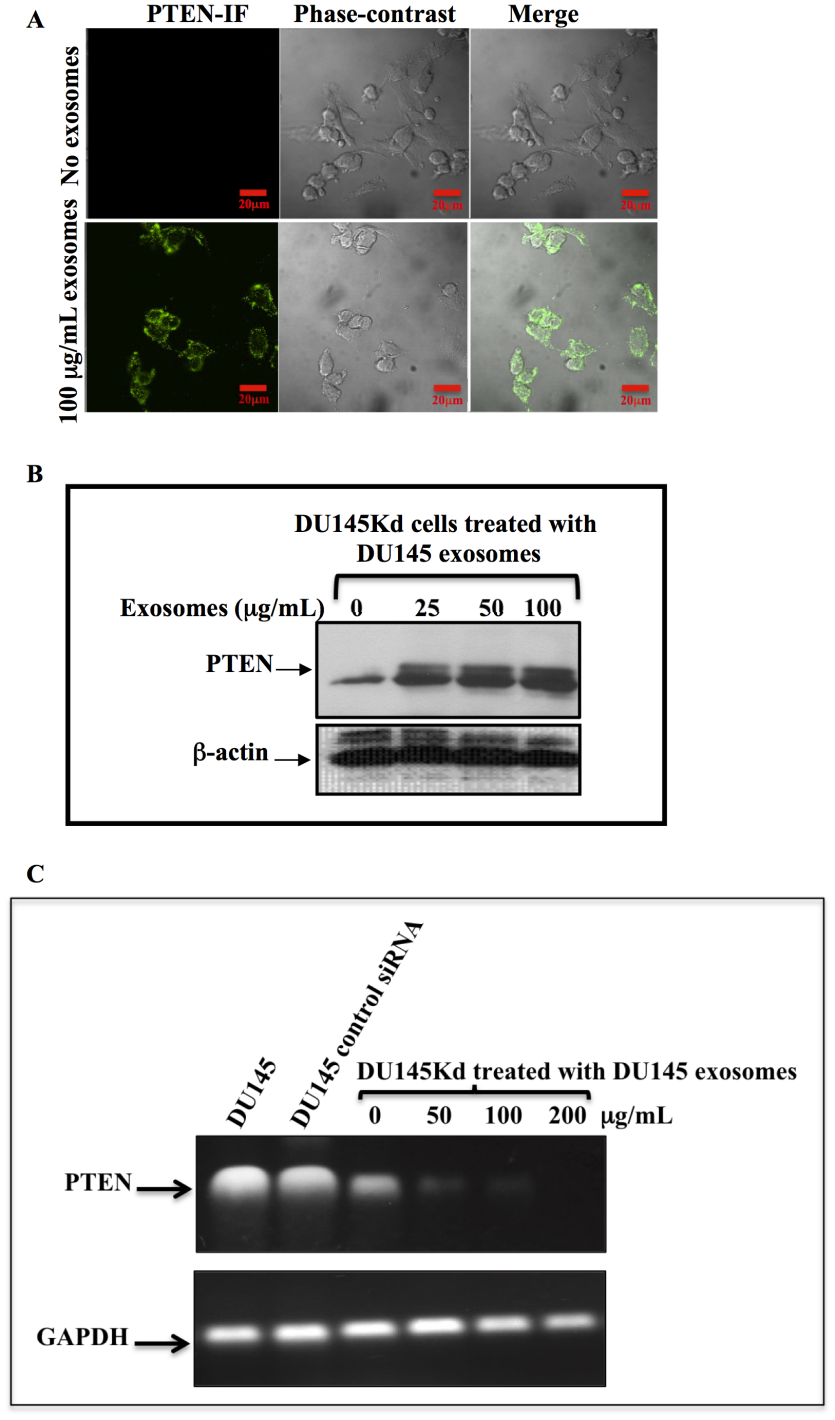
Exosomes transfer PTEN between prostate cancer cells. DU145Kd cells were incubated with different concentrations of exosomes derived from DU145 cells for 24 hours. A. DU145Kd cells were cultured in slide chambers and treated with DU145-derived exosomes. The cells were washed twice with phosphate buffered saline (PBS), and immunocytochemistry was performed with PTEN primary antibodies and Alexa fluor 488 secondary antibodies. The cells were visualized using confocal microscopy (IF- immunofluorescence). DU145Kd cells acquired PTEN (green) after incubation with Du145-derived exosomes. B. DU145Kd were cultured in 100-mm dishes and treated in the same manner as in (A) with different concentrations of DU145-derived exosomes. The cells were washed with PBS, lysed with RIPA lysis buffer, and analyzed for PTEN expression using immunobloting. DU145Kd cells acquired PTEN expression. C. Exosomes have an inhibitory effect on the transcription of PTEN. DU145Kd were treated with different concentrations of exosomes derived from DU145 parental cells. Total RNA was collected from the treated cells, DU145 cells, and DU145 with control siRNA. RT-PCR was performed using primers specific for PTEN. GAPDH was used as a control. RT-PCR was performed using a one-step RT-PCR kit. The products were resolved on a 1.1% agarose gel. D. Exosomes transfer PTEN to U87 PTEN^−/−^ cells. U87 cells were cultured in slide chambers and treated with DU145-derived exosomes. The cells were stained with PTEN antibodies and Alexa fluor 488 secondary antibodies, and then were visualized using a confocal microscope (IF-immunofluorescent). U87 cells, which do not express PTEN as they have mutations in both PTEN alleles, acquired PTEN expression (green).

To investigate the intercellular exchange of PTEN, exosomes derived from native DU145 PC cells were collected using the standard procedure of centrifugation. DU145Kd cells were incubated with the exosome preparation derived from parental DU145 cells. The apparent uptake of microvesicular PTEN by DU145Kd cells was observed using immunocytochemistry (background subtraction was performed using the untreated cells as control, and the fluorescence was subtracted from both nontreated and treated cells to show the acquisition of PTEN in exosome treated cells), and immunoblotting (Figure 2 A and B).

To ensure that the increase of PTEN in DU145Kd cells post-incubation with DU145-derived exosomes did not result from the stimulation of PTEN transcription by exosomes, we performed RT-PCR for PTEN in DU145Kd cells treated with different concentrations of DU145-derived exosomes. We observed an inhibitory effect on PTEN transcription (Figure 2C). To further ensure that we were observing the intercellular transfer of PTEN protein, as opposed to the stimulation of transcription, we used the glioblastoma cell line U87, a null genotype, which contains a mutation in both PTEN alleles [33]. When treated with exosomes derived from DU145 cells, U87 cells tested positive for PTEN (Figure 2D). These data confirm that the acquired PTEN in DU145Kd and U87 is solely related to the intercellular transfer of PTEN protein through exosome exchange. In addition, we treated the prostate cancer cell line PC-3, which is PTEN null, with exosomes derived from DU145 cells, showing the acquisition of PTEN (Figure S1). We determined PTEN expression in exosomes from different cancer cell lines i.e. lung carcinoma (A549), breast cancer (MDA-MB-231), colorectal carcinoma (HCT116), and pancreas adenocarcinoma (BxPC-3) (Figure S2, A), to show that PTEN is shed through exosomes from other cancer cell types. We also treated PC-3 cells with exosomes derived from HCT116 colorectal carcinoma cells, and found that the PC-3 cells acquired PTEN expression (Figure S2, B).

### Exosomes Transfer Active PTEN between Cancer Cells

**Figure 3:**
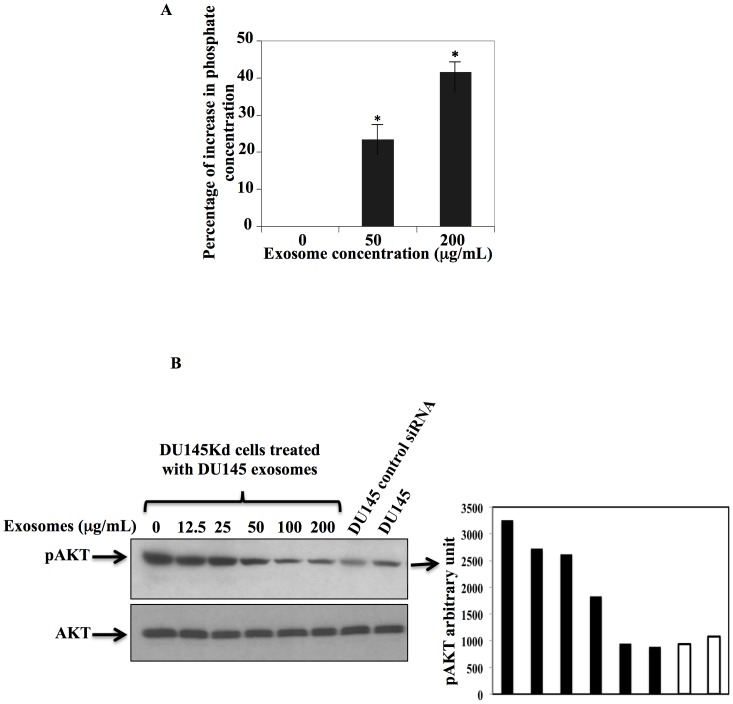
Exosomes transfer active PTEN to acceptor cells. A. DU145Kd cells were treated with exosomes derived from DU145 cells. PTEN was immunoprecipitated with PTEN antibodies, and PTEN phosphatase activity was assessed using the water-soluble substrate DiC8PtdIns (3, 4, 5) P3 (Echelon). The released free phosphates were measured with BIOMOL Green reagent and normalized against a reaction containing only PIP3 substrate. The results represent the average of three experiments ± SEM, and they are significant at P<0.01. B. PTEN-positive exosomes (from DU145) caused a decrease in AKT phosphorylation in acceptor cells (DU145Kd); AKT phosphorylation decreased to a level comparable to DU145 cells with control siRNA, and the parental counterpart DU145 cells (last two lanes to the right, respectively). C. DU145Kd cells were plated in 100-mm cell culture dishes and treated with different concentrations of DU145-derived exosomes. The cells were lysed and analyzed by immunobloting with p27 and cyclin D1 antibodies. PTEN induced the expression of p27 and reduced the expression of cyclin D1 (C). The two events led to the cells entering into cell-cycle arrest. The expression was normalized to β-actin.

To assess whether the PTEN transferred through exosomes is active, we treated DU145Kd cells with different concentrations of exosomes derived from DU145 cells. The treated cells were subjected to immunoprecipitation for PTEN. The immunoprecipitates were assessed for PTEN activity using a lipid-phosphatase assay. DU145Kd cells showed a substantial increase in phosphatase activity upon treatment with exosomes derived from DU145 cells (Figure 3A).

To investigate whether the transferred PTEN is competent to alter cell signaling in acceptor cells, DU145Kd cells were treated with exosomes derived from DU145 cells for 24 hours. The phosphorylation status of phosphatidylinositol 3′-kinase/serine-threonine kinase (AKT), the main substrate for PTEN, was assessed. In DU145Kd cells, we found a decrease in the phosphorylation of AKT that reached levels comparable to DU145 parental cells (PTEN positive) and DU145 cells transfected with control siRNA (Figure 3B).

To determine whether the transferred PTEN affects downstream cell signaling pathways associated with AKT, we assessed cyclin-dependent kinase (CDK) inhibitor p27 (KIP1) expression. p27 regulates cell proliferation, cell motility, and apoptosis. A reduction in p27 expression is observed in most lethal epithelial cancers and is associated with poor patient outcomes [34]. pAKT negatively regulates p27 to support antiapoptotic activity in cancer progression. We found that p27 expression is increased in DU145Kd cells when treated with DU145-derived exosomes (Figure 3C). It has been reported that G1 cell-cycle arrest is coordinated by PTEN lipid phosphatase activity through the upregulation of p27 and by PTEN protein phosphatase activity through the downregulation of cyclin D1 [35]. We determined that cyclin D1 is downregulated in DU145Kd cells treated with Du145 exosomes, as anticipated (Figure 3C).

### PTEN Transferred through Exosomes Affects Biological Functions in the Acceptor Cells

**Figure 4:**
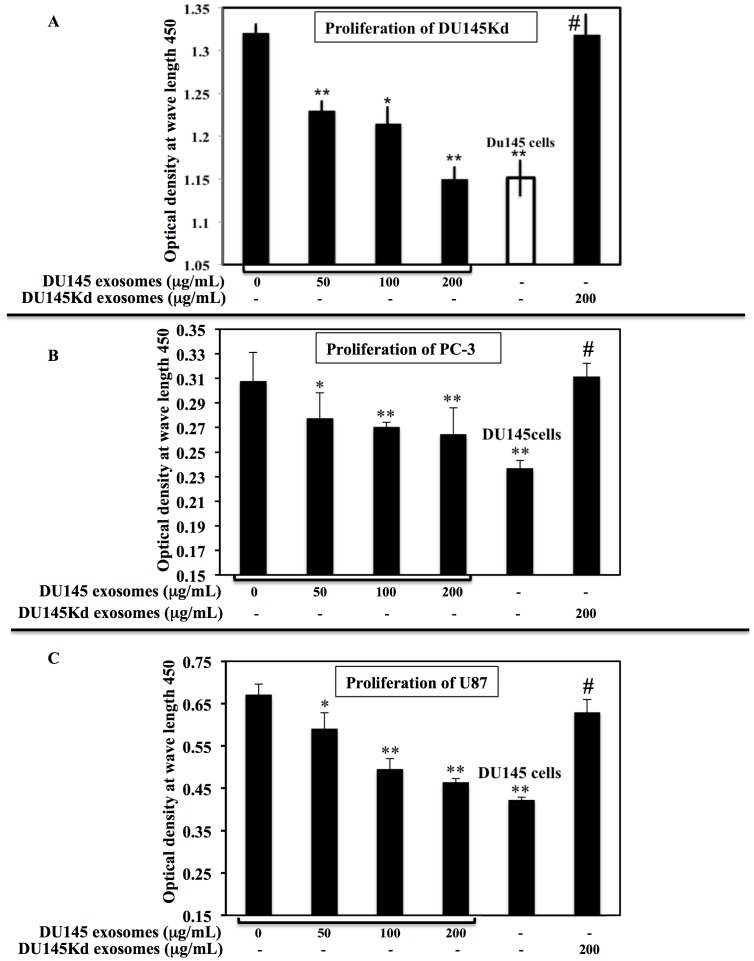
PTEN enriched exosomes modulate proliferation of cells lacking PTEN expression. Three cell lines lacking PTEN expression, DU14Kd, PC-3, and U87 (A, B and C, respectively), were treated with different concentrations of DU145 exosomes. A proliferation assay was performed using an assay kit. In all three cell lines, proliferation rates were inhibited in a concentration-dependent manner. The proliferation rates of DU145Kd cells reached a level comparable to DU145 cells, showing that exosomes completely compensated for the loss of PTEN (A). Exosomes derived from DU145Kd, which lack PTEN, showed no effect on cell proliferation of all three cell lines. The results are shown as the average of three experiments ± SEM. The differences from the control (0 exosomes) are significant * P<0.05, **P<0.01, and # the differences are not significant.

To assess whether the biochemical changes observed in Figure 3 are associated with modification of biological function, we assessed the effect of PTEN-positive exosomes (derived from DU145 cells) on the proliferation of three cell lines that lack PTEN expression. DU145Kd, PC-3, and U87 cells were treated with different concentrations of DU145 exosomes, and proliferation rates were inhibited in all three cell lines in a dose-dependent manner (Figure 4 A, B and C). Exosomes lacking PTEN expression, derived from DU145Kd, showed little effect on the proliferation rates.

### Incorporation of PTEN into Exosomes is Regulated by Oncogenes

**Figure 5:**
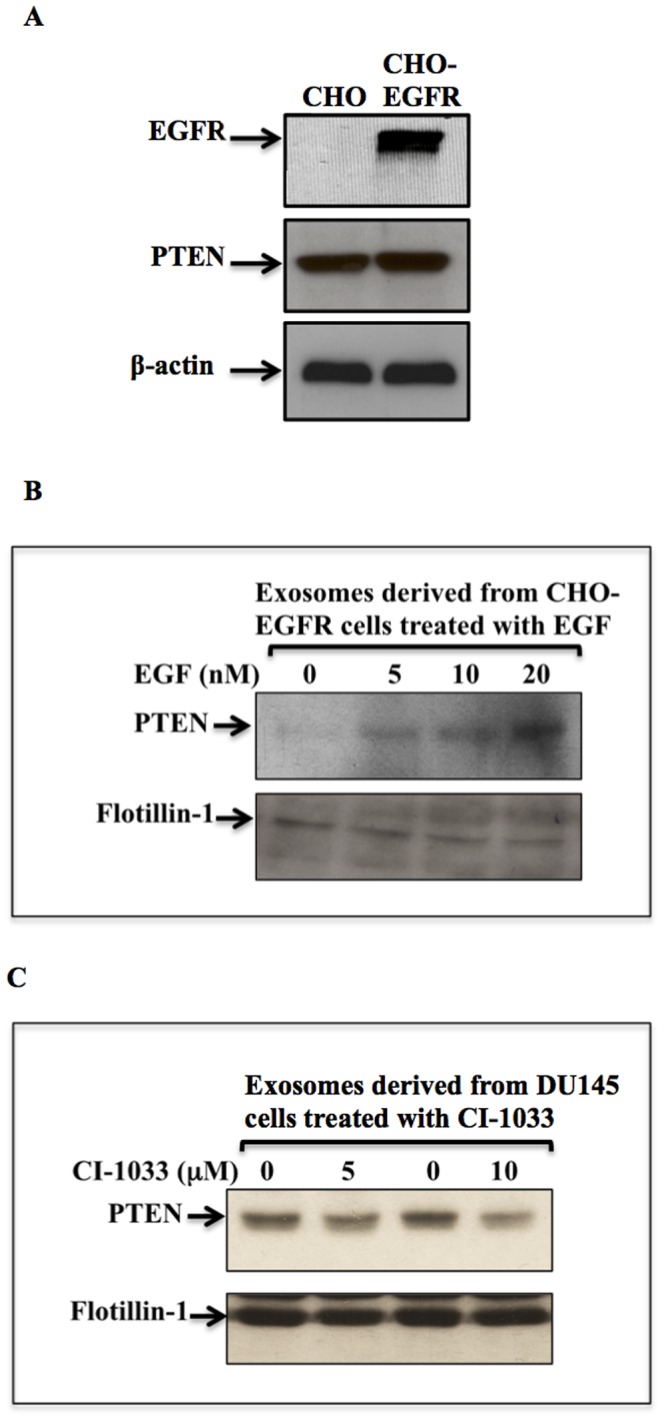
PTEN incorporation into exosomes is regulated by oncogenic molecules. A. Introducing EGFR in CHO cells showed no effect on the expression of PTEN in cells and exosomes. B. Stimulation of CHO-EGFR cells with the indicated concentrations of EGF (5, 10 and 20 ng/mL) led to an increase in PTEN incorporation in exosomes. C. DU145 cells were treated with two concentrations (5 µM, 10 µM) of CI-1033, an irreversible inhibitor of EGFR. PTEN incorporation into exosomes was inhibited in a concentration dependent manner; Flotilin-1 was used as a loading control for exosome concentration.

To investigate the mechanism of incorporation of PTEN into exosomal cargo, we tested the role of the oncogenic receptor Epidermal Growth Factor Receptor (EGFR) in this process. We used the Chinese Hamster Ovary (CHO) cell line, which are spontaneously immortalized [36] and have no EGFR expression. CHO cells stably transfected to express EGFR had similar levels of PTEN expressed in exosomes as the parental CHO cells (Figure 5A). Upon stimulation of CHO-EGFR cells with different concentrations of EGF (5, 10 and 20 ng/mL), there was an increase in the amount of PTEN in exosomes, in a concentration dependent manner (Figure 5B). In addition, we tested the effect of inhibiting EGFR in DU145 cells on PTEN incorporation into the exosomes. We treated the cells with the EGFR inhibitor CI-1033 (5, 10 µM). Inhibiting EGFR decreased the incorporation of PTEN into exosomes in a concentration dependent manner (Figure 5C).

### PTEN Status in PC Patients can be Assessed Using Blood Exosomes

**Figure 6:**
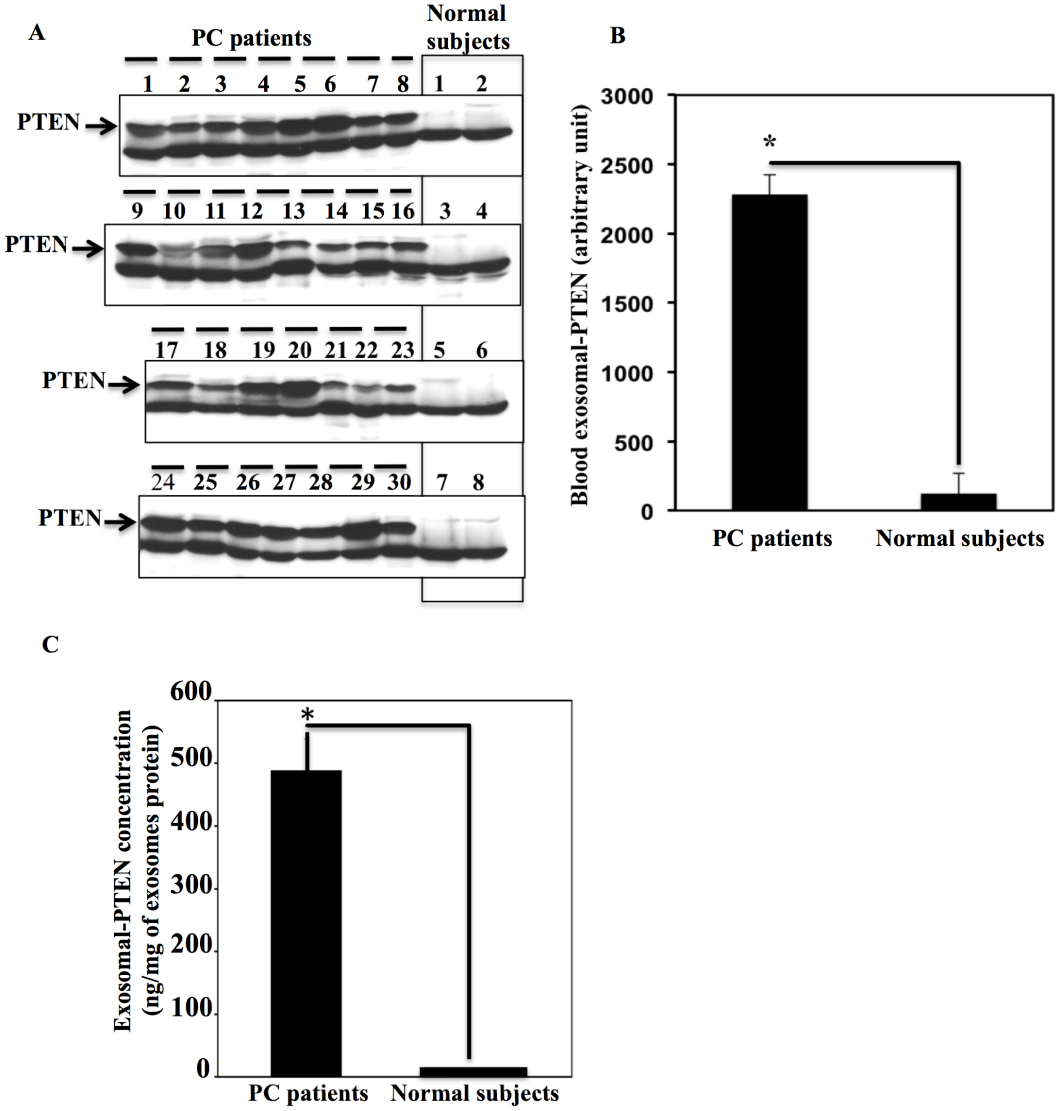
PTEN is detected in exosomes derived from the plasma of PC patients, but not normal subjects. A. Exosomes were collected from the plasma of 30 pre-prostatectomy PC patients and 8 normal subjects. Exosomes were subjected to standard immunoblotting analysis for the status of PTEN. The figure shows the ability of exosomes to assess the status of PTEN. As shown in the figure, the healthy subjects had no PTEN expression in their plasma exosomes. B. Optical densities for PTEN bands (A) were assessed using Quantity-1 software. Exosomal-PTEN expression in PC patients and normal subjects was calculated as the average ± SEM, and the differences between the two groups were highly significant (P<0.001). C. PTEN concentration in the exosomes from PC patient blood was assessed by immunoblotting standard concentrations of recombinant PTEN together with the patient samples. The results are the average of PTEN expression ± SEM and are statistically significant P<0.01.

To study the role of exosomes in the assessment of PTEN status in PC patients, we collected blood from 30 PC patients prior to prostatectomy, and 8 normal subjects matching the age of the patients (50–65 years). The normal volunteers had no history of cancer or previously documented elevated serum PSA. The blood was fractionated, and the plasma was used as a source for exosomes. PTEN-exosomes were immunoprecipitated using PTEN antibodies and Sepharose protein-G. The incorporation of PTEN into exosomes from PC patients’ blood was determined by immunoblotting, and different levels of expression were detected among patients. Interestingly, we found PTEN expression in exosomes from all PC patients, and no PTEN expression in the exosomes from the blood of the normal subjects (Figure 6 A, B); t-test analysis showed the results were highly significant P<0.001. In addition, we could assess the concentration of PTEN in PC patients’ exosomes by immunoblotting with standard concentrations of recombinant PTEN, and the concentration of PTEN was determined as (ng of PTEN/mg of exosome protein) (Figure 6C).

### Blood exosomal-PTEN Provides a Simple Tool to Assess PTEN Status

**Figure 7:**
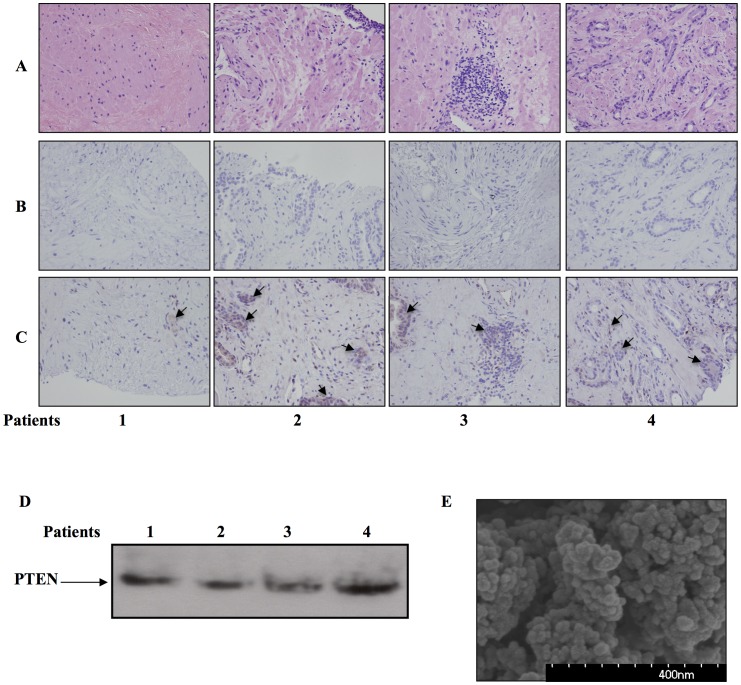
Exosomes provide a noninvasive, quantitative method to assess PTEN status in PC patients. This figure provides a comparison study of PTEN assessment using exosomal PTEN, and Immunohistochemistry of PC patient tumors. Immunohistochemistry of tumor tissues from four PC patients demonstrates the heterogeneity of PTEN expression in prostate tumors. A. Eosin & hematoxylin staining. B. Staining with secondary antibodies. C. Staining with PTEN specific antibodies, arrows indicate PTEN positive cells. D. Assessment of PTEN expression using plasma exosomes from the same patients. E) A scanning electron microscope micrograph showing the homogenous population of exosomes collected from the patient blood. Exosomes were attached to a cover slip using polylysine, and processed for scanning electron microscopy.

PC tumors are heterogeneous in PTEN expression as seen in (Figure 7 A, B, C). PTEN status in the tumors of four PC patients was determined by immunohistochemistry. The figure demonstrates the difficulty of forming a conclusion about PTEN expression using this technique. PTEN expression in the blood exosomes of the same patients is assessed in Figure 7D, demonstrating a simple and direct way to assess PTEN concentration in PC patients. Figure 7E is a scanning electron micrograph showing the homogenous population of blood exosomes.

### Expression of PSA in Exosome Preparations from PC Patients and Normal Subjects

**Figure 8:**
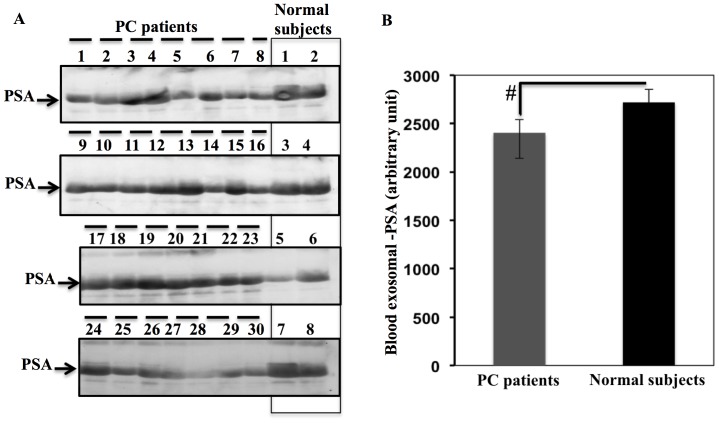
PSA is detected in exosomes derived from the plasma of PC patients and normal subjects. A. Exosomes were collected from the plasma of 30 pre-prostatectomy PC patients and 8 normal subjects. Exosomes were subjected to standard immunoblotting analysis for the status of PSA. As shown in the figure, all PC patients and healthy subjects were positive for exosomal PSA. B. Optical densities of PSA bands (A) were assessed using Quantity-1 software. Exosomal-PSA expression was calculated as the average ± SEM, and the differences between the two groups were found to be non-significant.

Using 30 PC patients and 8 normal subjects, we detected PSA in exosomes from both PC patients and normal subjects (Figure 8A): optical densities of the PSA bands (from Figure 8A) were used for statistical analysis. The differences in exosomal PSA between PC patients and normal subjects were not significant (Figure 8B). Detection of PSA in exosomes from normal subjects and PC patients points to a new fraction of PSA, which may be related to the lack of specificity associated with the PSA test. We assessed PSA status because it is the traditional biomarker used in PC diagnosis. We wanted to determine whether PSA is expressed on patients’ blood exosomes, and assess the differences in expression between PC patients and normal subjects. This allowed us to compare the expression pattern of PSA and exosomal-PTEN in PC patients and normal subjects. We performed this comparison to assess the specificity of exosomal-PTEN compared to PSA, the traditional prostate cancer biomarker.

## Discussion

An important observation made during the present study is that PTEN is expressed in exosomes derived from cancer cells, and it is not detected in exosomes from normal cells, although the normal cells themselves express PTEN. Thus, the incorporation of PTEN in exosomes may represent a characteristic of cancer cells not found in normal cells. This finding may suggest an exclusionary mechanism used by cancer cells to downregulate PTEN. This new mechanism requires further investigation to characterize the molecules responsible for directing PTEN to exosomes. Because this mechanism is detectable in cancer cells and we didn’t detect it in normal cells, we hypothesized that it might be under the control of oncogenes. Therefore, we studied the effect of introducing EGFR in CHO cells. Introducing EGFR in these cells showed no effect on exosomal-PTEN (Figure 5A), however when the introduced EGFR was activated by EGF treatment we saw an increase in exosomal-PTEN expression (Figure 5B). In addition, we are studying molecules associated with PTEN in exosomes to identify possible chaperone molecules regulated by cancer cells to direct PTEN to exosomes. Recently, it has been reported that Ndfip1, an adaptor protein for members of the Nedd4 family of E3 ubiquitin ligases, has a role in directing PTEN to exosomes [29]. Decoding this mechanism may provide a unique opportunity to target cancer cells.

This study also shows the effect of PTEN-expressing exosomes derived from cancer cells on modulating the cell proliferation of recipient cells with reduced or null PTEN expression. The incubation of PTEN-expressing exosomes with cells that have decreased PTEN expression (DU145Kd cells), or cells with no PTEN expression (U87 cells), leads to the uptake of PTEN by these cells. Upon uptake of PTEN-enriched exosomes, the acceptor cells acquired significantly higher PTEN activity, demonstrated by a PTEN-activity assay. In addition, PTEN activity is evidenced by the decreased phosphorylation of AKT, which is a reflection of the dephosphorylation of PIP3 and PIP2 by PTEN [37], [38], [39]. Furthermore, it is reported that PTEN coordinates cell-cycle arrest in G1 by downregulating cyclin D1 via PTEN protein phosphatase activity, and upregulating p27 via PTEN lipid phosphatase activity [35]. We found that p27 is upregulated by exosomal-PTEN transfer, and that cyclin D1 is downregulated. Taken together, these findings imply that PTEN derived from exosomes is biologically active and can modulate cell growth and proliferation.

Our study suggests that the source of the PTEN observed in DU145Kd and U87 cells is related to the transfer of PTEN from PTEN-positive cells via exosomes. This is evidenced by the fact that exosomes have an inhibitory effect on the transcription of PTEN, as demonstrated by RT-PCR (Figure 2C). This inhibitory effect may be explained by the expression of miRNA 21 in exosomes, which negatively regulates transcription of PTEN in acceptor cells [40]. In the transfer experiments using U87 cells, PTEN was acquired from transferred exosomal-PTEN, as U87 cells have mutations in both PTEN alleles resulting in a null genotype [33]. Both approaches indicate that the presence of PTEN is solely related to PTEN transfer via exosomes.

PTEN has a prognostic value for tumor recurrence and metastasis in PC patients [20], [21], [22]. In these studies, tissue samples from the tumor were used to assess PTEN expression through immunohistochemistry by comparing tumor tissue staining with cells positive or negative for PTEN expression [20], [21]. In our experience, it is very difficult to derive a conclusion about PTEN status using immunohistochemistry due to the heterogeneity of PTEN expression in the tumor tissue (Figure 7 A, B, C). The challenge of studying PTEN and any other marker of solid tumors is the need for direct access to the tumor through biopsies. This problem is especially challenging in clinical trials involving potential cancer therapies, as enrolled patients need to be exposed to multiple biopsies [41]. At this point, the only way to assess the expression of PTEN and other molecules in PC patients is by direct access to tumor tissue, through biopsies or after radical prostatectomy. In this study, we propose a new noninvasive tool to profile PC tumors through blood exosomes. The expression of PSA in exosomes has been reported in exosomes from the urine of PC patients [42]. The expression of PSA in exosomes from the blood of PC patients is an indicator that exosomes are derived from the diseased prostate gland. However, at this stage, we cannot rule out the possibility that PSA is incorporated into exosomal cargo after PSA secretion to the blood stream, as it has been suggested that PSA binds other blood proteins in the circulation and only a small fraction of PSA is not bound [43]. The incorporation of PSA in exosomal cargo should be addressed in future studies. In this context, exosomes may provide a new tool to overcome the lack of specificity and prognostic value of PSA reported by Tosoian & Loeb [24], by correlating PSA expression to other proteins expressed in exosomes, such as PTEN (in this study) or other molecules.

From our data, PTEN appears to be a better indicator than PSA in characterizing PC and can discriminate between PC patients and normal subjects, although this finding still needs validation and confirmation. The existence of tumor derived exosomes in the circulation of PC patients may be due to the fact that tumor vasculature is poorly organized and highly permeable [44]. This may prevent tumor cells from re-uptaking shed exosomal-PTEN, by eliminating the accumulation of these exosomes within the tumor microenvironment and surrounding tissues.

## Supporting Information

Figure S1 **PTEN null PC-3 prostate cancer cells acquire PTEN through exosomes derived from DU145 cells.** PC-3 cells were cultured in slide chambers and treated with DU145-derived exosomes. The cells were washed three times with phosphate buffered saline (PBS), and immunocytochemistry was performed with PTEN primary antibodies and Alexa -fluor 488 secondary antibodies. The cells were visualized using confocal microscopy (IF- immunofluorescence). PC-3 cells acquired PTEN (green) after incubation with the exosomes.
(TIFF)

Figure S2 **Different cancer cell types express PTEN in their exosomes, and transfer PTEN to other cells through exosomes.** Exosomes were collected from different cancer cell types, i.e. lung carcinoma (A549), breast cancer (MDA-MB-231), colorectal carcinoma (HCT116), and pancreas adenocarcinoma (BxPC-3). Exosomes were profiled for PTEN expression by immunoblotting, and were positive for PTEN expression (A). PC-3 PTEN-null cells were treated with exosomes derived from HCT116 cells, and acquired PTEN expression as shown by immunocytochemistry (B).
(TIFF)
