# Supplementary figures and images for: Regulation of the Tumor Suppressor PTEN through Exosomes: A Diagnostic Potential for Prostate Cancer
Source: PLoS One. 2013 Jul 25;8(7):e70047. doi: 10.1371/journal.pone.0070047 (PMC3723640; doi:10.1371/journal.pone.0070047)

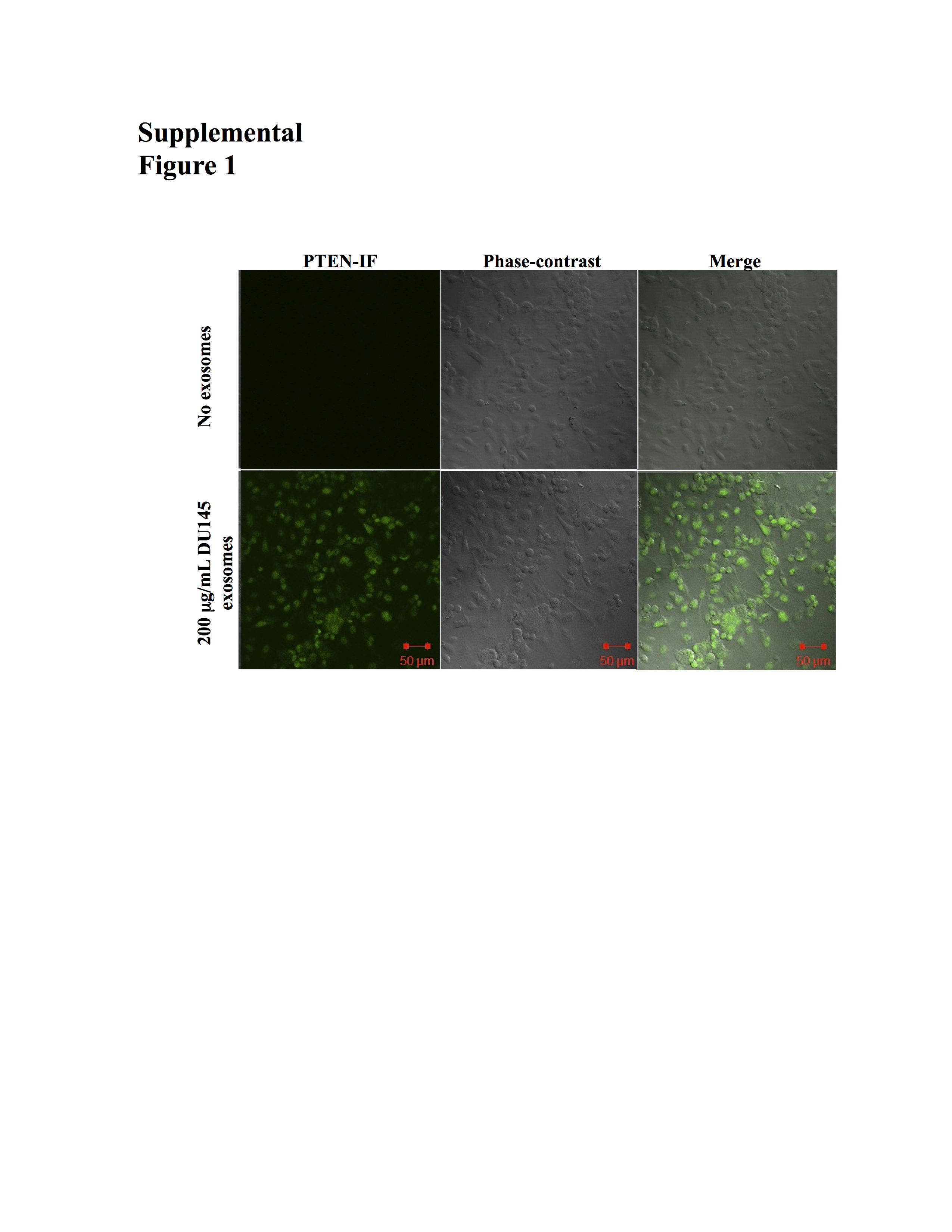

Supplement: Figure S1 — PTEN null PC-3 prostate cancer cells acquire PTEN through exosomes derived from DU145 cells. PC-3 cells were cultured in slide chambers and treated with DU145-derived exosomes. The cells were washed three times with phosphate buffered saline (PBS), and immunocytochemistry was performed with PTEN primary antibodies and Alexa -fluor 488 secondary antibodies. The cells were visualized using confocal microscopy (IF- immunofluorescence). PC-3 cells acquired PTEN (green) after incubation with the exosomes. (TIFF) [file pone.0070047.s001.tiff]

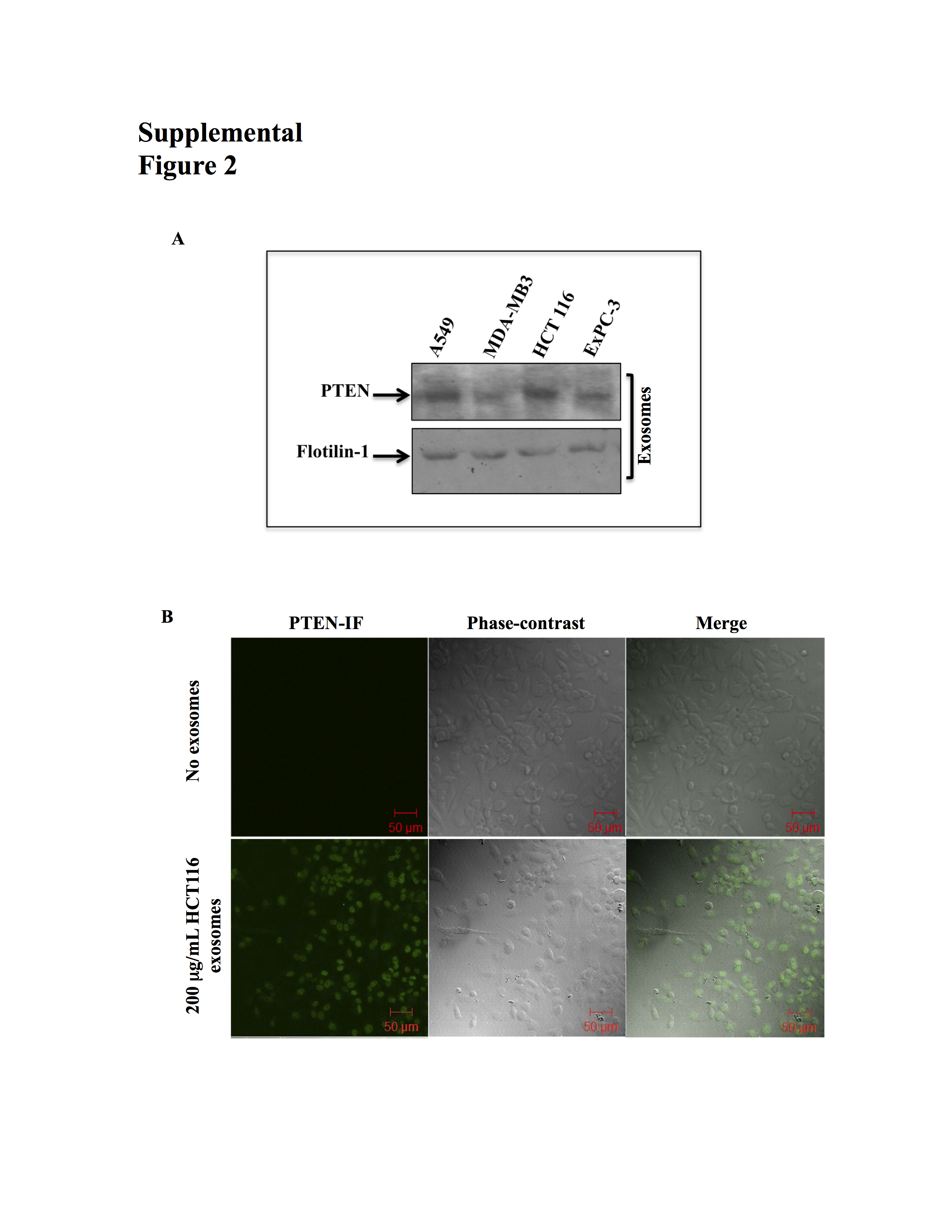

Supplement: Figure S2 — Different cancer cell types express PTEN in their exosomes, and transfer PTEN to other cells through exosomes. Exosomes were collected from different cancer cell types, i.e. lung carcinoma (A549), breast cancer (MDA-MB-231), colorectal carcinoma (HCT116), and pancreas adenocarcinoma (BxPC-3). Exosomes were profiled for PTEN expression by immunoblotting, and were positive for PTEN expression (A). PC-3 PTEN-null cells were treated with exosomes derived from HCT116 cells, and acquired PTEN expression as shown by immunocytochemistry (B). (TIFF) [file pone.0070047.s002.tiff]
